# Monocytes and Macrophages in COVID-19

**DOI:** 10.3389/fimmu.2021.720109

**Published:** 2021-07-21

**Authors:** Rainer Knoll, Joachim L. Schultze, Jonas Schulte-Schrepping

**Affiliations:** ^1^ Systems Medicine, Deutsches Zentrum für Neurodegenerative Erkrankungen (DZNE), Bonn, Germany; ^2^ Genomics & Immunoregulation, Life & Medical Sciences (LIMES) Institute, University of Bonn, Bonn, Germany; ^3^ PRECISE Platform for Single Cell Genomics and Epigenomics, Deutsches Zentrum für Neurodegenerative Erkrankungen (DZNE) and the University of Bonn, Bonn, Germany

**Keywords:** monocytes, macrophage, COVID-19, SARS-CoV-2, hyperinflammation, scRNA-seq, alveolar macrophage, viral infection

## Abstract

COVID-19 is a contagious viral disease caused by SARS-CoV-2 that led to an ongoing pandemic with massive global health and socioeconomic consequences. The disease is characterized primarily, but not exclusively, by respiratory clinical manifestations ranging from mild common cold symptoms, including cough and fever, to severe respiratory distress and multi-organ failure. Macrophages, a heterogeneous group of yolk-sac derived, tissue-resident mononuclear phagocytes of complex ontogeny present in all mammalian organs, play critical roles in developmental, homeostatic and host defense processes with tissue-dependent plasticity. In case of infection, they are responsible for early pathogen recognition, initiation and resolution of inflammation, as well as repair of tissue damage. Monocytes, bone-marrow derived blood-resident phagocytes, are recruited under pathological conditions such as viral infections to the affected tissue to defend the organism against invading pathogens and to aid in efficient resolution of inflammation. Given their pivotal function in host defense and the potential danger posed by their dysregulated hyperinflammation, understanding monocyte and macrophage phenotypes in COVID-19 is key for tackling the disease’s pathological mechanisms. Here, we outline current knowledge on monocytes and macrophages in homeostasis and viral infections and summarize concepts and key findings on their role in COVID-19. While monocytes in the blood of patients with moderate COVID-19 present with an inflammatory, interferon-stimulated gene (ISG)-driven phenotype, cellular dysfunction epitomized by loss of HLA-DR expression and induction of S100 alarmin expression is their dominant feature in severe disease. Pulmonary macrophages in COVID-19 derived from infiltrating inflammatory monocytes are in a hyperactivated state resulting in a detrimental loop of pro-inflammatory cytokine release and recruitment of cytotoxic effector cells thereby exacerbating tissue damage at the site of infection.

## Introduction

COVID-19 (1, 2) is primarily a mild to moderate respiratory tract infection caused by severe acute respiratory syndrome coronavirus 2 (SARS-CoV-2), an enveloped, single-stranded RNA betacoronavirus (3–5). While 80% of the infections lead to asymptomatic or mild disease with common cold symptoms including dry cough, headache, loss of taste, dyspnea, fatigue and fever, contained by an efficient immune response (6–8), 15% of the patients go on to develop severe disease requiring intensive care and oxygen support and 5% develop critical disease with life-threatening pneumonia, acute respiratory distress syndrome (ARDS) and septic shock often culminating in multi-organ dysfunction and death (9).

Age, various comorbidities, including diabetes, obesity, lung and cardiovascular diseases, as well as genetic polymorphisms correlate with a higher risk of respiratory failure (10–13).

SARS-CoV-2, similar to SARS-CoV (14), enters host cells *via* the angiotensin-converting enzyme 2 (ACE2) receptor and uses the human protease TMPRSS2 as entry activator (15, 16). These genes are expressed in a wide range of cells, including nasal and bronchial epithelial cells, enterocytes, cardiomyocytes, vascular and testicular cells, placental trophoblasts, bile duct cells (17, 18) as well as macrophages (19, 20). Furthermore, additional entry molecules, such as Neuropilin (NRP1), have been discussed to facilitate viral cell entry (21, 22).

Although acute respiratory manifestations are the most common feature, COVID-19 can have multiple acute extra-pulmonary clinical effects likely to be related to vascular pathology (23), and also long-lasting complications referred to as the post-COVID syndrome or long COVID, including fatigue or neurological sequelae (24–27).

Control of viral infections and resolution of inflammation generally depends on dose and route of infection, viral virulence properties as well as host immune factors (28, 29). Tightly regulated interactions between epithelial cells and immune cells, orchestrated by cytokine signaling and direct cellular contacts, play a critical role, also in COVID-19 (30, 31). Moreover, viral clearance does not necessarily mean recovery to a healthy state. Hyperactivated and dysregulated immune cells pose a substantial danger for exacerbated tissue damage (32–34) and alter susceptibility to secondary bacterial superinfection (35, 36).

Severe COVID-19 has been associated with pronounced changes in peripheral immune activity (37, 38), including increased levels of acute phase reactants and pro-inflammatory cytokines (39, 40), neutrophilia and emergence of immature and low-density neutrophils (41, 42), increased neutrophil-to-lymphocyte ratio and lymphopenia (43) as well as myeloid inflammation (44) and reduced expression of the human leukocyte antigen DR isotype (HLA-DR) by circulating monocytes (42, 45).

A time-dependent, multi-stage disease model for COVID-19 has been proposed (28). Early and efficient activation of the immune system through induction of a potent interferon response is crucial for controlling the virus. However, a delayed and/or prolonged interferon response may lead to progressive tissue damage, which may ultimately result in a deleterious hyperinflammation characterized by excessive activation of mononuclear phagocytes (MNPs) and coagulation in combination with dysregulation of tissue repair mechanisms and fibrosis (46).

Together with dendritic cells (DC), macrophages and monocytes form the MNP system (47). In addition to being professional antigen-presenting cells (APC), MNPs sense and phagocytose pathogens, mediate leukocyte recruitment, initiate and shape immune responses and regulate inflammation.

Macrophages are a heterogeneous family of tissue-resident, phagocytic innate immune cells, including brain microglia, liver Kupffer cells and lung alveolar and interstitial macrophages, that play an important role in tissue homeostasis and immune defense (48). In case of infection, macrophages sense danger signals from microbial pathogens or tissue damage *via* a plethora of pattern recognition receptors (PRRs), and respond by release of inflammatory molecules that eliminate pathogens, initiate inflammation and recruitment of additional effector cells and promote tissue repair (32). However, as is the case for example in macrophage activation syndrome (MAS), an overwhelming macrophage response can be detrimental to the host (33).

Monocytes are blood-circulating, phagocytic innate immune cells classically divided into three subsets based on their respective expression of CD14 and CD16 [classical (CD14^+^CD16^−^), non-classical (CD14dimCD16^+^), and intermediate (CD14^+^CD16^+^)] (48, 49). Under pathological conditions, including viral infections, monocytes, activated and recruited by inflammatory mediators, infiltrate affected tissues and acquire inflammatory macrophage and DC-like phenotypes to fulfil their effector functions of pro- and anti-inflammatory activities, antigen-presentation and tissue remodeling (50).

Here, we outline major findings concerning the role of monocytes and macrophages in COVID-19 and put them into the context of general knowledge of these cells in viral infections.

## Alveolar and Interstitial Macrophage Ontogeny and Function

Every day, the lung inhales thousands of liters of air containing high amounts of pathogens including viruses, bacteria, and fungi (51). To prevent infection and its resulting complications for the organism, a tight control by the immune system is needed. In the lung, macrophages are the most abundant immune cell type under homeostatic conditions. Based on their exact location, they can be separated in at least two different populations; the interstitial macrophages (IMs) and alveolar macrophages (AMs) (52, 53).

IMs reside in the parenchyma between the microvascular endothelium and alveolar epithelium, while AMs have close contact to epithelial cells of alveoli and reside in the airspace lumen. However, a recent study by Neupane et al. showed that AMs are, in contrast to macrophages in other tissues, not sessile but can crawl in and between alveoli using the pores of Kohn (54). By expression of integrins, CD11c^neg^CD11b^pos^ IMs can be distinguished from CD11c^pos^CD11b^neg^ AMs (52).

In addition to mucus and the epithelial barrier, AMs are the first defenders against pathogens entering the respiratory system. They originate from the yolk sac and populate the lung early after birth (55, 56). AMs have proliferative capacity, thus can persist over the lifespan by self-renewal and are independent of replacement from the bone marrow (57–59). AMs detected in bronchoalveolar lavage fluid (BALF) after lung transplantation were almost exclusively donor derived (60). Following depletion of lung macrophages in mice, repopulation occurred almost entirely by *in situ* proliferation (61). In contrast, analysis of pulmonary MNPs in patients receiving bone marrow transplantation for hematologic disorders provided evidence for replenishment of AMs by monocytes of bone marrow origin (62). The current understanding of the plastic composition and complex ontogeny of pulmonary MNPs is best described by a dynamic interplay of cells derived from yolk sac macrophages, fetal liver, and adult monocytes given pathologic threats and vacant niches (63).

The functional phenotype of AMs strongly depends on the local microenvironment and can change with contact with epithelial cells, oxygen tension and surfactant-rich fluid, highlighting the relevance of AM plasticity (64, 65). Therefore, AMs can be pro-/anti-inflammatory, pro-/anti-fibrotic, pro-asthmatic, pro-resolving and/or tissue-reparative. In the physiological state, AMs are critical for homeostasis by removing apoptotic cells, foreign materials, and surfactant, thereby ensuring that the lungs remain free of debris. Of note, they typically show an immunosuppressive phenotype (52). The anti-inflammatory program is critical to prevent unwanted inflammation in the lung that can be of serious danger for the organism. Although AMs have antigen presenting capacities and express HLA-DR, they promote tolerance and suppress lymphocyte activation under homeostatic conditions by producing immunosuppressive prostaglandins and TGFβ, of which the latter together with retinoic acid may drive the development of FOXP3^+^ regulatory T cells (Treg), further strengthening the anti-inflammation (66–68). By signaling through various receptors, such as by CD200R (69), SIRPα (70), mannose receptor CD206 (71), MACRO (72), TREM2 (73), and soluble mediators including Interleukin (IL)-10 (74), TGFβ (75) and PPARγ (76) AMs experience negative regulation. For instance, CD200 is expressed on the luminal side of respiratory epithelial cells and binding to CD200R on AMs leads to the suppression of pro-inflammatory genes in AMs (69).

Upon lung injury or infection, AMs can mount inflammatory responses (77). Destruction of airway epithelium can lead to a loss of exposure to regulatory ligands, such as CD200, resulting in a switch to a pro-inflammatory program in AMs (69). Recognition of pathogen associated molecular patterns (PAMP) of invading pathogens by AMs *via* PRRs further enhances this activation. These activated AMs are characterized by enhanced phagocytic capacity, higher oxidative burst and increased release of pro-inflammatory cytokines and chemokines, which results in inflammation and recruitment of other immune effector cells to the lung, including neutrophils (78). Recruited cells also include monocytes, which can differentiate into macrophage and DC-like cells, thus often referred to as monocyte-derived AMs (Mo-AMs) and DC (Mo-DC), upon arrival in peripheral tissues and can further enhance inflammation (79, 80). Their different ontogeny and functionality can influence the outcome of infection and inflammation.

Importantly, prolonged, and dysregulated inflammation caused by macrophages and monocytes can cause collateral tissue damage (81). To prevent prolonged inflammation and to limit tissue damage and fibrosis, AMs have evolved several strategies. These include phagocytosis of dying cells, e.g. neutrophils (82) preventing the release of their pro-inflammatory and toxic contents and triggering the secretion of TGFβ, IL-10, prostaglandin E2 and platelet-activating factor from AMs (83).

Respiratory pathologies such as asthma, chronic obstructive pulmonary disease (COPD), cystic fibrosis and idiopathic pulmonary fibrosis (IPF) are characterized by defective AM phagocytosis resulting in continuous inflammation (84–87).

Besides respiratory pathologies, cigarette smoking also presents a major risk factor for impaired AM function. AMs of smokers are expanded in numbers compared to non-smoking controls but show less phagocytic activity, glucose oxidation rate and cytokine production compared to non-smoking controls, which increases the risk of severe disease progression upon bacterial and viral infection (88–91).

After a successful inflammation, suppressive stimuli as described above are restored and AMs shift to an anti-inflammatory, tissue reparative phenotype restoring the homeostasis of the lung (65).

## The Role of Lung Macrophages in Viral Respiratory Infections

As described above, the lung is at permanent risk of infection by several pathogens, amongst them viruses such as rhinovirus, respiratory syncytial virus, influenza virus and coronavirus. Despite their obvious relevance, investigation of human lung MNPs during respiratory infections has been limited so far and most of our knowledge comes from animal models. For instance, Schneider et al. showed that AM-depleted WT mice infected with influenza A virus had impaired gas exchange and fatal hypoxia (92). Similar results were obtained in pigs which, after AM depletion by dichloromethlyene diphosphonate, were infected with seasonal human H1N1 influenza virus resulting in 40% mortality rate and increased suffering from severe respiratory signs, whereas infected control pigs showed less severe symptoms with no mortality (93).

Notably, various viruses, including Influenza, Chikungunya, human herpes and Zika virus, have been shown to utilize monocytes and macrophages as vessels for virus replication, dissemination, or long-term persistence within tissues. They enter the cells through endocytosis, phagocytosis, macropinocytosis or membrane fusion and induce elevated expression of proinflammatory signaling and antiviral molecules (94–99). Direct infection of macrophages with SARS-CoV has also been shown, which, however, did not lead to dissemination or virus amplification but rather to an impaired type I interferon (IFN) response potentially worsening disease outcome (100).

Upon viral infection, AMs produce high levels of cellular mediators, including IL-1β, CCL3, CCL7 and CCL2, also known as monocyte chemotactic protein 1 (MCP1), which rapidly recruits CCR2-expressing bone marrow-derived monocytes into the lung. Furthermore, AMs are the main producers of type I IFN to trigger an antiviral response in influenza infection (101, 102). Of note, type I IFN production by AMs was higher than by plasmacytoid DCs (pDCs), coined as the natural “IFN producing cells”, in response to virus, indicating that pDCs may play a subordinate role in the defense against viral infections in the lung (102). Moreover, alveolar epithelial cells also did not produce any type I IFN in response to influenza, further stressing the key role of AMs (103). Type I IFNs can signal autocrine and paracrine resulting in the activation of antiviral transcriptional programs including the transcription of ISG such as *ISG15*, *IFIT1* and *STAT2*, which can suppress viral replication (104, 105). Interestingly, not all virus infections trigger an increased type I IFN response. For instance, when human AMs were infected with coronavirus strain 229E (HCoV-299E), they secreted increased amounts of TNF, CCL5 and CCL4 (MIP-1β), causing inflammation, but IFN-β levels remained unchanged (106).

Viral infection triggers the migration of circulating monocytes to the lung guided by pro-inflammatory cytokines, such as CCL2 and CCL3, increasing the number of defending mononuclear phagocytes and enhancing inflammation (79). This is a necessary defense response, since viruses such as influenza can either reduce the numbers of resident AMs dramatically or impair their phenotype. When BALB/c mice were infected with influenza, 90% of resident AMs were lost in the first week after infection (107). This, however, was strain specific, since C57B1/6 mice did not show loss of AMs but rather an impaired phenotype. Nevertheless, both consequences were driven by IFN-γ and resulted in increased susceptibility to bacterial superinfections leading to significant body weight loss and mortality. Furthermore, a recent study by Neupane et al. showed that crawling of AMs, which is critical for AM function, was impaired after influenza infection. Again, this impairment was mediated by the IFN-γ pathway and resulted in increased risk for bacterial superinfections (54).

## The Role of Monocytes and Alveolar Macrophages in COVID-19

### The Involvement of Monocytes and Macrophages in SARS-CoV-2 Induced Hyperinflammation

COVID-19 is characterized by a systemic increase of numerous cytokines, including IL-1α, IL-1β, IL-6, IL-7, tumor necrosis factor (TNF), type I and II IFN, and the inflammatory chemokines CCL2, CCL3 and CXCL10 (40, 108, 109). Elevated levels of CCL2 and CCL7, two chemokines potent at the recruitment of CCR2^+^ monocytes, have also been found in BALF from patients with severe COVID-19 (110).

The term “cytokine storm”, historically described as an influenza-like syndrome that occurred after systemic infections and immunotherapies (111), has quickly become widely used, both in scientific publications and the media, to describe the cytokine response in COVID-19 (39). Although the increased systemic cytokine response in COVID-19 is undisputed, the term “cytokine storm” in COVID-19 pathophysiology is a topic of debate, as TNF, IL-6, and IL-8 concentrations in COVID-19 are less strong compared to sepsis, acute respiratory distress syndrome unrelated to COVID-19, trauma, cardiac arrest, and cytokine release syndrome (CRS) (112–115). Moreover, COVID-19 immune responses are highly dynamic as shown by time-dependent alterations of the systemic levels of many cytokines including IL-6 (40). Considering the co-occurrence of distinct systemic pro-inflammatory cytokine waves with the emergence of aberrant and immunosuppressive innate immune cells further complicates the exact terminology of immunopathology in severe COVID-19 and suggests a much more complex host-pathogen interaction better described by the term viral sepsis (28). In any case, the systemic cytokine profile observed in patients suffering from severe COVID-19 does resemble those observed in CRS, such as macrophage activation syndrome (MAS), which led early on to the working hypothesis that dysregulated activation of the MNP compartment contributes to COVID-19-associated hyperinflammation (33, 113).

The induction of cytokine production in MNPs in COVID-19 can either be triggered *via* recognition of damage-associated molecular patterns (DAMPS) released from epithelial cells affected by SARS-CoV-2 by PRRs or by direct recognition of viral pathogen-associated molecular patterns (PAMPs) *via* specific Toll-like receptors, i.e. TLR2 and TLR4, the retinoic acid-inducible gene I (RIG-I) or the melanoma differentiation associated gene (MDA)-5 (116–119). Furthermore, C-type lectin receptors, including DC-SIGN, L-SIGN, LSECtin, ASGR1, CLEC4K (Langerin) and CLEC10A (MGL), as well as Tweety family member 2 have been identified to interact with the SARS-CoV-2 spike protein inducing proinflammatory responses, but not allowing direct infection. Notably, however, these interactions were shown to promote virus transfer to ACE^+^ cells (120, 121).

SARS-CoV-2 infection of lung-resident MNPs might result either from phagocytosis of infected alveolar epithelial cells followed by viral escape from the lysosome or by direct infection. *In vitro* experiments with human monocyte-derived DC and macrophages with SARS-CoV-2 have demonstrated that both cell types are permissive to SARS-CoV-2 as measured by quantification of SARS-CoV-2 nucleocapsid protein expression after *in vitro* infection, but did not support productive viral replication. Interestingly, expression of proinflammatory cytokines and chemokines however was only triggered in macrophages and not DC under these experimental conditions (122). Additional independent infection experiments confirmed the abortive SARS-CoV-2 infection in human monocyte-derived DC and macrophages *in vitro* and corroborated the induction of antiviral and proinflammatory cytokines, including IFN-α/β, TNF, IL-1β, -6, and -10, as well as CXCL10, leading to type I IFN–mediated host cell death (123). Accordingly, investigation of cell tropism and immune activation profiles of SARS-CoV-2 in *ex vivo* organ cultures of human lung tissues revealed infection of type I and II pneumocytes as well as AMs (124), confirmed by detection of SARS-CoV-2 in AMs in autopsy samples from COVID-19 patients (125). Interestingly, analysis of murine AMs derived from human (h)ACE2 transgenic animals revealed different susceptibility to SARS-CoV-2 infection depending on their cytokine-induced polarization as *in vitro* treatment with IFN-γ and LPS caused increased infection rates compared to pre-treatment with IL-4 (126). Furthermore, *in vitro* treatment of PMA-differentiated THP-1 human macrophages and isolated CD14^+^ monocytes with SARS-CoV-2 spike protein after LPS stimulation exposed a hyperresponsiveness to TLR signals by suppression of IRAK-M (127). Moreover, antibody-dependent mechanisms of infection present a conceivable alternative pathway and have been described for SARS-CoV (128, 129). Besides this body of evidence demonstrating the induction of inflammatory pathways in monocytes and macrophages upon recognition of SARS-CoV-2, metabolic alterations in these cells have been reported. *Ex vivo* infected human monocytes shifted their metabolism and became highly glycolytic leading to elevated glucose levels promoting SARS-CoV-2 replication and cytokine production (130). Moreover, monocytes derived from COVID-19 patients were shown to have increased lipid droplet accumulation, which was explained by the modulation of lipid synthesis and uptake investigated using *in vitro* infection models and again favored virus replication and inflammatory mediator production (131). Interestingly, the pharmacological inhibition of DGAT1, a key enzyme in lipid droplet formation, inhibited SARS-CoV-2 replication and production of pro-inflammatory mediators presenting a new opportunity for therapeutic intervention.

Corresponding to the systemic increase of cytokine and chemokine levels, quantitative and qualitative changes in immune cell populations, particularly in the myeloid compartment, have been observed in blood and lungs of patients with COVID-19 dependent on disease severity.

Flow cytometric analyses of peripheral blood reported reduced percentages of total monocytes in the blood of severe COVID-19 cases (38, 132, 133). Notably, this reduction was observed only transiently in a longitudinal study of immune cells in severe cases pointing to the highly time-sensitive immune response (134).

Beyond quantitative changes, striking disease-specific differences in monocyte phenotypes in the blood and monocyte–macrophage composition in the lung have been consistently reported. A significant expansion of CD14^+^CD16^+^ monocytes featuring high expression of IL-6 in the blood discriminated patients with COVID-19 admitted to ICUs from those who did not require intensive care (132). Moreover, significantly reduced numbers of non-classical and intermediate monocytes are found in acute patients with symptoms of severe SARS-CoV-2 infection (135) and circulating classical monocytes show clear signs of activation, including increased expression of CD169 (135). In addition, experimentally infected monocytes and those from patients with severe COVID-19 requiring intensive care feature inflammasome activation and increased pyroptosis associated with caspase-1 activation (136). Furthermore, increased proliferation of monocytes derived from patients with severe COVID-19 after *in vitro* challenge with lipopolysaccharide was discussed as an indicator for a release of immature myeloid cells from the bone marrow reminiscent of emergency myelopoiesis (137) and contributing to innate immune dysfunction (138). Most prominently and consistent across all studies, reduced HLA-DR expression on monocytes – a well-established marker of immune suppression – was reported in patients suffering from severe COVID-19 (41, 42, 134, 139, 140). Decreased HLA-DR expression appeared to be strongly associated with COVID-19 disease severity, exemplified by lower expression of HLA-DR by monocytes in patients admitted to the ICU versus non-ICU patients (140) and in non-survivors versus survivors (141). Furthermore, the presence of HLA-DR^lo^ monocytes in severe cases of COVID-19 was found to be positively correlated with levels of the soluble immunosuppressive factors IL-10, TGF-β, VEGFA, and AREG (142). In addition, reduced HLA-DR and CD86 expression together with elevated levels of IL-1β, IL-6, IL-8, IL-10, IL-17 and IFN-γ were observed in children with multisystem inflammatory syndrome (MIS-C) associated with SARS-CoV-2 infection (143). Downregulation of HLA-DR is a molecular feature often described for monocytic myeloid-derived suppressor cells (MDSC) – a cellular state of monocytes described to develop during chronic inflammation, especially late-stage cancers, and defined by T cell immunosuppressive functions (144). Functional assessment of HLA-DR^-^ monocytes derived from COVID-19 patients indeed confirmed their capacity to suppress T cell proliferation, partly *via* ARG-1, and thus supports the MDSC state beyond phenotypic description (145). Interestingly, the HLA-DR^-^ monocytes specific for severe acute COVID-19 have furthermore been found to express CPT1, an enzyme essential for fatty acid oxidation, again highlighting the relevance of immunometabolic effects of SARS-CoV-2 infection (146).

### High-Resolution Single-Cell Omics Characterization of Monocytes and Macrophages in the Blood and Lungs of COVID-19 Patients

Application of high-resolution omics technologies with single-cell resolution, which were only developed and became widely applied within the last decade, has confirmed their great potential to rapidly decipher the immune response to an emerging pathogen during the COVID-19 pandemic. The first transcriptomic immune atlas of circulating peripheral blood mononuclear cells (PBMC) from 10 COVID-19 patients demonstrated globally decreased lymphocyte counts, while inflammatory myeloid cells were found to be more abundant (147). By now, at least 16 other studies have used scRNA-seq to characterize the immune response to SARS-CoV-2 (31, 41, 42, 45, 108, 148–158). While initial studies were based on low sample numbers limiting their explanatory power, latest reports comprised samples derived from more than 100 individuals, included longitudinal samples or profiled matched samples from multiple tissues. Single-cell transcriptomic analysis of PBMC in 7 hospitalized COVID-19 patients revealed a depletion of CD16^+^ monocytes in peripheral blood and the induction of an ISG signature in CD14^+^ monocytes, but detected no substantial induction of pro-inflammatory cytokine genes, such as TNF, IL6, IL1β, CCL3, CCL4 or CXCL2 in these cells, suggesting that peripheral monocytes are no major contributors to the cytokine response in COVID-19 (155). The lack of expression of inflammatory cytokines in innate immune cells in the periphery of COVID-19 patients was confirmed by multiplex plasma cytokine analysis, mass cytometry, and scRNA-seq in a cohort of 76 COVID-19 patients and 69 healthy individuals from two cohorts. Despite significantly upregulated levels of inflammatory molecules in the plasma of COVID-19 patients and transiently induced expression of ISGs in peripheral immune cells, an impaired cytokine response in blood myeloid cells and pDCs, with markedly reduced expression of IL-6, TNF and IL-1β upon TLR stimulation, was observed emphasizing a tissue origin of the plasma cytokines (108). Interestingly, the lack of ISG-expressing cells associated with mild disease was linked to severe disease-specific production of antibodies suppressing cellular interferon responses (159). In a dual-center, two-cohort study, we combined scRNA-seq and single-cell proteomics of whole-blood and PBMC and determined changes in the immune cell composition and activation in mild versus severe COVID-19 over time. While non-classical monocyte numbers were diminished in COVID-19, HLA-DR^hi^CD11c^hi^ inflammatory monocytes with an ISG signature were elevated in mild COVID-19 and monocytes in severe COVID-19 featured strongly reduced HLA-DR expression, high expression levels of genes with anti-inflammatory and immature properties, including SELL (CD62L), CD163, MPO and PLAC8, as well as increased expression of S100A family members, e.g. S100A12 (42). Loss of non-classical monocytes, reduced HLA-DR expression in monocytes and massive release of S100A family members was observed in severe cases of COVID-19 in multiple additional studies (41, 151, 156, 157), albeit disease stratification into mild, moderate, severe and critical disease showed slight differences. In addition, calprotectin (S100A8/S100A9) plasma levels and decreased frequencies of non-classical monocytes were found to discriminate patients who develop a severe form of COVID-19 (41).

Although the analysis of blood was extremely instructive particularly when assessing systemic effects of COVID-19, the lung presents the primary site of infection for SARS-CoV-2 and investigating the local immune system response is key to understanding the pathology. Activated monocytes of the blood have been shown to infiltrate the lungs in patients with COVID-19 and in animal models of SARS-CoV-2 infection (160, 161). In their seminal study, Liao et al. characterized BALF from patients with varying severity of COVID-19 and healthy individuals using scRNA-seq and reported striking shifts in cellular composition with increased proportions of macrophages and neutrophils and lower proportions of DCs and T cells in samples from severe/critical COVID-19 compared to those from moderate disease and healthy individuals. Within the MNP compartment, they observed a depletion of tissue-resident AMs and a replacement by inflammatory monocyte-derived macrophages in patients with severe disease. Notably, cytokine and chemokine expression levels differed dependent on disease severity. While CXCL9, CXCL10 and CXCL11 expression levels were increased both in moderate and severe disease compared to healthy levels, IL1β, IL6, TNF as well as CCL2, CCL3, CCL4 and CCL7 were expressed at higher levels in lung macrophages from patients with severe COVID-19. CXCL16, which interacts with the chemokine receptor CXCR6 and attracts subsets of T cells, was specifically induced in patients with moderate disease. These distinct expression profiles suggest that lung macrophages in patients with severe COVID-19 may promote tissue infiltration of inflammatory monocytes enhancing local inflammation, whereas macrophages in patients with moderate COVID-19 preferentially attract T cells. Furthermore, macrophage subpopulations specific for severe disease presented with immunoregulatory features but also expression of the profibrotic genes TREM2, TGFB2, and SPP1 (45). In agreement with this study, scRNA-seq data of nasopharyngeal and bronchial samples from 19 COVID-19 patients revealed the presence of inflammatory non-tissue resident and monocyte-derived macrophages expressing various cytokines, including IL1, TNF, CCL2 and CCL3, as well as enhanced interactions between epithelial and immune cells as determined by ligand–receptor expression profiling, in critical compared to moderate disease (31). Interestingly, comparing macrophages from the lower to the upper airways demonstrated increased expression of inflammatory cytokines and chemokines in the bronchia. Furthermore, monocyte-to-macrophage trajectory analysis in scRNA-seq of BALF samples from COVID-19 patients exposed enrichment of chronic hyperinflammatory monocytes in critical COVID-19 presenting with elevated expression levels of inflammasome-related genes (NLRP3, IL1-β, IL10RA) and genes associated with fibrosis (FGL2, TGFB1, COTL1) potentially contributing to tissue damage in severe COVID-19 (154). Single-nucleus (sn)RNA-seq on lung autopsies from 19 COVID-19 decedents confirmed the lungs to be highly inflamed with dense infiltration of aberrantly activated monocyte-derived macrophages and alveolar macrophages in the tissue (153). Another cross-sectional scRNA-seq of 780,000 PBMC sampled from 130 patients collected across three medical centers in the UK revealed the presence of a non-classical monocyte population characterized by the expression of complement transcripts C1QA/B/C in COVID-19. The complement system is a key host-defense mechanism with capacity to exacerbate tissue injury through its proinflammatory effects. Notably, integration of these PBMC transcriptomes with data derived from BALF samples (45) followed by partition-based graph abstraction (PAGA) analysis demonstrated transcriptional similarity between the circulating C1QA/B/C^+^CD16^+^ monocytes and alveolar macrophages in COVID-19 emphasizing the altered composition of the lung MNP compartment (150). The consistent reports of aberrant CD163^hi^ and HLA-DR^lo^ monocyte populations expressing the chemokine receptor CCR2 in the blood and hyperactivated airway monocytes and macrophages producing pro-inflammatory chemokines, including CCL2 and CCL3, were furthermore confirmed by high-dimensional phenotypic, transcriptomic, and functional profiling of immune cells from paired airway and blood samples obtained longitudinally from patients with severe COVID-19 (149).

**Figure 1:**
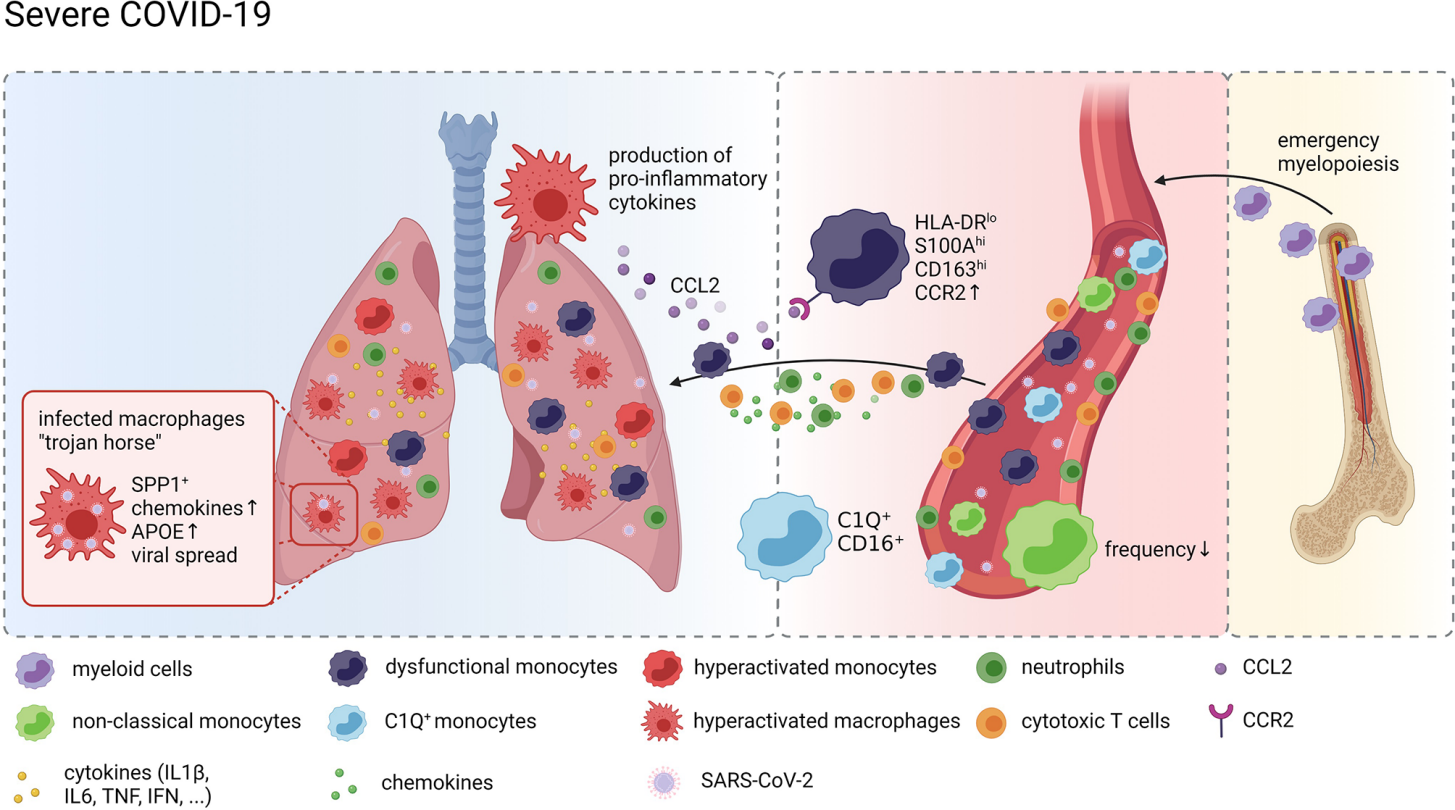
Monocytes and Macrophages in COVID-19. Graphical overview of the compositional and molecular alterations in monocyte and alveolar macrophage populations in COVID-19 created with BioRender.com. Distinct monocyte and macrophage phenotypes were identified in the peripheral blood of patients with severe COVID-19 including immature cells indicating emergency myelopoiesis, dysfunctional HLA-DR^lo^ classical monocytes and complement gene expressing non-classical monocytes. These cells are attracted to the lung by pro-inflammatory chemokines resulting in a continuous accumulation of hyperactivated MNPs producing more pro-inflammatory mediators recruiting more inflammatory cells, including cytotoxic T cells and neutrophils, thus further exacerbating inflammation and tissue damage. SARS-CoV-2 infected macrophages in the lung may act as trojan horses propagating SARS-CoV-2 infection and spreading hyperinflammation across the lung.

Taken together, these data strongly suggest a model of a vicious cycle of pro-inflammatory cytokine release by hyperactivated lung MNPs resulting in erratic infiltration of pro-inflammatory effector cells, including dysregulated monocytes and cytotoxic T cells, which in turn exacerbates tissue damage and fuels macrophage activation (**Figure 1**).

### Detection of SARS-CoV-2 RNA in Single-Cell RNA Profiles of Monocytes and Macrophages

Since SARS-CoV-2 exploits the host cell transcriptional machinery to express viral genes, viral transcripts can be detected alongside human mRNA transcripts in scRNA-seq data, thereby allowing for identification of infected cells and their unique properties at single-cell resolution. Bost et al. developed a new computational pipeline, called Viral-Track, to quantify viral RNA in single-cell transcriptomic data. Application of their approach to scRNA-seq data of BALF from the aforementioned study by Liao et al. revealed the presence of viral reads in samples derived from patients with severe, but not mild disease, suggestive of a differential viral load in the lung (162). The highest levels of viral RNA were observed in ciliated and epithelial progenitor cells. However, viral RNA was also detected in a subset of macrophages characterized by expression of SPP1. Whether these transcripts resulted from direct infection of and viral replication within the myeloid cells or whether the cells phagocytosed cellular material carrying viral RNA could not be clarified by this approach. However, the results of the single-cell specific viral RNA quantification allowed for differential gene expression in infected *vs* bystander SPP1^+^ macrophages, which revealed increased expression of chemokines (CCL7, CCL8, and CCL18) and APOE in virus-positive cells. The approach was further advanced by Wauters et al. who stratified SARS-CoV-2 infected cells in scRNA-seq data from BALF samples derived from patients with mild and critical COVID-19 by the presence of viral transcripts from distinct viral open reading frames (ORF). Detection of spike protein (S) specific transcripts in epithelial cells and consequentially reduced expression of ISGs suggests that S^+^ epithelial cells have actively been infected. In contrast, transcripts of the nucleocapsid protein (N) and the ORF10 and ORF1a were detected in myeloid and lymphoid cells at much higher levels than in epithelial cells. Comparing N^+^ *vs* N^-^ alveolar and monocyte-derived macrophages determined genes involved in MHC class-II expression and ISG to be upregulated in response to the virus. Grant et al. followed an alternative approach to answer whether SARS-CoV-2 productively infects myeloid cells. Adding the negative-strand SARS-CoV-2 transcripts, which are transiently formed during viral replication, to the reference genome during alignment and quantification of their single-cell and bulk BALF transcriptome data allowed for evaluation of replicating SARS-CoV-2 in AMs. Besides the expected detection of positive and negative strand transcripts in epithelial cells, viral reads were also detected in subsets of macrophages suggesting that AMs harbor SARS-CoV-2 and allow viral replication *in vivo* (158), challenging the results on abortive infection gained from *in vitro* experiments. Interestingly, immunostaining of post-mortem tissue from patients who had died from COVID-19 revealed the presence of SARS-CoV-2 nucleoprotein in and the expression of ACE2 on populations of CD169^+^ macrophages in lymph nodes and the spleen (20). Given the increasing body of evidence in support of active infection of and the indication of productive viral replication in AMs by SARS-CoV-2, Grant et al. have come up with the hypothesis that AMs may act as a Trojan horse, transferring the virus to adjacent lung regions, thereby slowly propagating SARS-CoV-2 infection and spreading hyperinflammation across the lung (**Figure 1**).

## Outlook and Open Questions

After more than a year into the pandemic, it is rather clear that the innate immune system and in particular monocytes and macrophages are linked to the heterogeneity of the COVID-19 disease courses. For example, HLA-DR^hi^ monocytes are typically seen in mild cases, while HLA-DR^lo^ S100^+^ cells dominate in severe COVID-19. Future work needs to untangle which molecular mechanisms are responsible for these different cellular responses. For example, are certain signals from the microenvironment normally increasing the induction of HLA-DR molecules missing in patients with severe disease course? Are elevated levels of inhibitory factors such as certain prostaglandins or TGFβ responsible for the molecular phenotype of MNPs in severe COVID-19. Furthermore, is there a direct link between fibrotic lung disease as a result of severe COVID-19 with ARDS and changes in the MNP compartment or other immune cells like NK cells. And if this is the case, are the anti-fibrotic molecular programs of monocytes and macrophages not working or do these cells suddenly gain pro-fibrotic functionality. Are molecular changes seen in these cells early during the disease predictive for disease courses leading to irreversible tissue damage as it is proposed for some patients with Long COVID-19? Even if the pandemic will be under control due to world-wide vaccination programs and other medical measures, the sequelae of Long COVID-19 and its potential burden on long-term health requires further studies into the role of the immune system, in particular the innate immune system with monocytes, macrophages and granulocytes requiring special attention.

## Author Contributions

All authors listed have made a substantial, direct, and intellectual contribution to the work, and approved it for publication.

## Funding

The work of JS was supported by the German Research Foundation (DFG) under Germany’s Excellence Strategy (EXC2151–390873048), the EU project SYSCID (grant number 733100), the BMBF-funded grant iTREAT (01ZX1902B), ERA CVD (grant number 00160389), and the BMBF-funded excellence project Diet–Body–Brain (DietBB) (grant number 01EA1809A).

## Conflict of Interest

The authors declare that the research was conducted in the absence of any commercial or financial relationships that could be construed as a potential conflict of interest.

## Abbreviations

AMs, alveolar macrophages; APC, antigen-presenting cells; COPD, chronic obstructive pulmonary disease; COVID-19, Coronavirus disease 2019; DC, dendritic cells; IL, intereukin; IFN, interferon; IPF, idiopathic pulmonary fibrosis; ISG, interferon-stimulated genes; MNPs, mononuclear phagocytes; Mo-AMs, monocyte-derived AMs; Mo-DC, monocyte-derived DC; ORF, open reading frames; SARS-CoV-2, severe acute respiratory syndrome coronavirus 2; scRNA-seq, single-cell RNA-sequencing; snRNA-seq, single-nucleus RNA-sequencing.

## References

[1] Berlin DA Gulick RM Martinez FJ. Severe Covid-19. N Engl J Med (2020) 383:2451–60. 10.1056/NEJMcp2009575 32412710

[2] Gandhi RT Lynch JB del Rio C. Mild or Moderate Covid-19. N Engl J Med (2020) 383:1757–66. 10.1056/NEJMcp2009249 32329974

[3] Kim D Lee JY Yang JS Kim JW Kim VN Chang H. The Architecture of SARS-CoV-2 Transcriptome. Cell (2020) 181:914–21.e10. 10.1016/j.cell.2020.04.011 32330414 PMC7179501

[4] Wu F Zhao S Yu B Chen YM Wang W Song ZG. A New Coronavirus Associated With Human Respiratory Disease in China. Nature (2020) 579:265–9. 10.1038/s41586-020-2008-3 PMC7094943 32015508

[5] Zhang YZ. Holmes EC. A Genomic Perspective on the Origin and Emergence of SARS-CoV-2. Cell (2020) 181:223–7. 10.1016/j.cell.2020.03.035 PMC7194821 32220310

[6] Huang C Wang Y Li X Ren L Zhao J Hu Y. Clinical Features of Patients Infected With 2019 Novel Coronavirus in Wuhan, China. Lancet (2020) 395:497–506. 10.1016/S0140-6736(20)30183-5 31986264 PMC7159299

[7] Thevarajan I Nguyen THO Koutsakos M Druce J Caly L van de Sandt CE. Breadth of Concomitant Immune Responses Prior to Patient Recovery: A Case Report of Non-Severe COVID-19. Nat Med (2020) 26:453–5. 10.1038/s41591-020-0819-2 PMC7095036 32284614

[8] Guan W Ni Z Hu Y Liang W Ou C He J. Clinical Characteristics of Coronavirus Disease 2019 in China. N Engl J Med (2020) 382:1708–20. 10.1056/nejmoa2002032 PMC7092819 32109013

[9] Fu L Wang B Yuan T Chen X Ao Y Fitzpatrick T. Clinical Characteristics of Coronavirus Disease 2019 (COVID-19) in China: A Systematic Review and Meta-Analysis. J Infect (2020) 80:656–65. 10.1016/j.jinf.2020.03.041 PMC7151416 32283155

[10] Zhang Q Liu Z Moncada-Velez M Chen J Ogishi M Bigio B. Inborn Errors of Type I IFN Immunity in Patients With Life-Threatening COVID-19. Science (2020) 370:eabd4570. 10.1126/science.abd4570 32972995 PMC7857407

[11] Beck DB Aksentijevich I. Susceptibility to Severe COVID-19. Science (2020) 370:404–5. 10.1126/science.abe7591 33093097

[12] Pairo-Castineira E Clohisey S Klaric L Bretherick AD Rawlik K Pasko D. Genetic Mechanisms of Critical Illness in COVID-19. Nature (2021) 591:92–8. 10.1038/s41586-020-03065-y 33307546

[13] Osuchowski MF Winkler MS Skirecki T Cajander S Shankar-Hari M Lachmann G. The COVID-19 Puzzle: Deciphering Pathophysiology and Phenotypes of a New Disease Entity. Lancet Respir Med (2021) 9(6):622–42. 10.1016/s2213-2600(21)00218-6 PMC8102044 33965003

[14] Drosten C Günther S Preiser W van der Werf S Brodt H-R Becker S. Identification of a Novel Coronavirus in Patients With Severe Acute Respiratory Syndrome. N Engl J Med (2003) 348:1967–76. 10.1056/nejmoa030747 12690091

[15] Shang J Wan Y Luo C Ye G Geng Q Auerbach A. Cell Entry Mechanisms of SARS-CoV-2. Proc Natl Acad Sci USA (2020) 117:11727–34. 10.1073/pnas.2003138117 PMC7260975 32376634

[16] Hoffmann M Kleine-Weber H Schroeder S Krüger N Herrler T Erichsen S. SARS-CoV-2 Cell Entry Depends on ACE2 and TMPRSS2 and Is Blocked by a Clinically Proven Protease Inhibitor. Cell (2020) 181:271–80.e8. 10.1016/j.cell.2020.02.052 32142651 PMC7102627

[17] Hikmet F Méar L Edvinsson Å Micke P Uhlén M Lindskog C. The Protein Expression Profile of ACE2 in Human Tissues. Mol Syst Biol (2020) 16:e9610. 10.15252/msb.20209610 32715618 PMC7383091

[18] Sungnak W Huang N Bécavin C Berg M Queen R Litvinukova M. SARS-CoV-2 Entry Factors are Highly Expressed in Nasal Epithelial Cells Together With Innate Immune Genes. Nat Med (2020) 26:681–7. 10.1038/s41591-020-0868-6 PMC8637938 32327758

[19] Song X Hu W Yu H Zhao L Zhao Y Zhao X. Little to No Expression of Angiotensin-Converting Enzyme-2 on Most Human Peripheral Blood Immune Cells But Highly Expressed on Tissue Macrophages. Cytom Part A (2020) 2020:1–10. 10.1002/cyto.a.24285 33280254

[20] Xiang Q Feng Z Diao B Tu C Qiao Q Yang H. SARS-CoV-2 Induces Lymphocytopenia by Promoting Inflammation and Decimates Secondary Lymphoid Organs. Front Immunol (2021) 12:661052. 10.3389/fimmu.2021.661052 33995382 PMC8113960

[21] Daly JL Simonetti B Klein K Chen KE Williamson MK Antón-Plágaro C. Neuropilin-1 Is a Host Factor for SARS-CoV-2 Infection. Science (2020) 370:861–5. 10.1126/science.abd3072 PMC7612957 33082294

[22] Cantuti-Castelvetri L Ojha R Pedro LD Djannatian M Franz J Kuivanen S. Neuropilin-1 Facilitates SARS-CoV-2 Cell Entry and Infectivity. Science (2020) 370:856–60. 10.1126/science.abd2985 PMC7857391 33082293

[23] Gupta A Madhavan MV Sehgal K Nair N Mahajan S Sehrawat TS. Extrapulmonary Manifestations of COVID-19. Nat Med (2020) 26:1017–32. 10.1038/s41591-020-0968-3 PMC11972613 32651579

[24] Fraser E. Long Term Respiratory Complications of Covid-19. BMJ (2020) 370:m3001. 10.1136/bmj.m3001 32747332

[25] Helms J Kremer S Merdji H Clere-Jehl R Schenck M Kummerlen C. Neurologic Features in Severe SARS-CoV-2 Infection. N Engl J Med (2020) 382:2268–70. 10.1056/NEJMc2008597 PMC7179967 32294339

[26] Carfì A Bernabei R Landi F. Persistent Symptoms in Patients After Acute COVID-19. JAMA - J Am Med Assoc (2020) 324:603–5. 10.1001/jama.2020.12603 PMC7349096 32644129

[27] Nalbandian A Sehgal K Gupta A Madhavan MV McGroder C Stevens JS. Post-Acute COVID-19 Syndrome. Nat Med (2021) 27:601–15. 10.1038/s41591-021-01283-z PMC8893149 33753937

[28] Schultze JL Aschenbrenner AC. COVID-19 and the Human Innate Immune System. Cell (2021) 184:1671–92. 10.1016/j.cell.2021.02.029 PMC7885626 33743212

[29] Rouse BT Sehrawat S. Immunity and Immunopathology to Viruses: What Decides the Outcome? Nat Rev Immunol (2010) 10:514–26. 10.1038/nri2802 PMC3899649 20577268

[30] Branchett WJ Lloyd CM. Regulatory Cytokine Function in the Respiratory Tract. Mucosal Immunol (2019) 12:589–600. 10.1038/s41385-019-0158-0 30874596 PMC7051906

[31] Chua RL Lukassen S Trump S Hennig BP Wendisch D Pott F. COVID-19 Severity Correlates With Airway Epithelium–Immune Cell Interactions Identified by Single-Cell Analysis. Nat Biotechnol (2020) 38:970–9. 10.1038/s41587-020-0602-4 32591762

[32] Merad M Martin JC. Pathological Inflammation in Patients With COVID-19: A Key Role for Monocytes and Macrophages. Nat Rev Immunol (2020) 20:355–62. 10.1038/s41577-020-0331-4 PMC7201395 32376901

[33] Schulert GS Grom AA. Pathogenesis of Macrophage Activation Syndrome and Potential for Cytokine-Directed Therapies. Annu Rev Med (2015) 66:145–59. 10.1146/annurev-med-061813-012806 PMC5846123 25386930

[34] Karki R Sharma BR Tuladhar S Williams EP Zalduondo L Samir P. Synergism of TNF-α and IFN-γ Triggers Inflammatory Cell Death, Tissue Damage, and Mortality in SARS-CoV-2 Infection and Cytokine Shock Syndromes. Cell (2021) 184:149–68.e17. 10.1016/j.cell.2020.11.025 33278357 PMC7674074

[35] Goulding J Godlee A Vekaria S Hilty M Snelgrove R Hussell T. Lowering the Threshold of Lung Innate Immune Cell Activation Alters Susceptibility to Secondary Bacterial Superinfection. J Infect Dis (2011) 204:1086–94. 10.1093/infdis/jir467 PMC3164429 21881124

[36] Oliver BGG Lim S Wark P Laza-Stanca V King N Black JL. Rhinovirus Exposure Impairs Immune Responses to Bacterial Products in Human Alveolar Macrophages. Thorax (2008) 63:519–25. 10.1136/thx.2007.081752 18245149

[37] Chen G Wu D Guo W Cao Y Huang D Wang H. Clinical and Immunological Features of Severe and Moderate Coronavirus Disease 2019. J Clin Invest (2020) 130:2620–9. 10.1172/JCI137244 PMC7190990 32217835

[38] Qin C Zhou L Hu Z Zhang S Yang S Tao Y. Dysregulation of Immune Response in Patients With Coronavirus 2019 (COVID-19) in Wuhan, China. Clin Infect Dis (2020) 71:762–8. 10.1093/cid/ciaa248 PMC7108125 32161940

[39] Mehta P McAuley DF Brown M Sanchez E Tattersall RS Manson JJ. COVID-19: Consider Cytokine Storm Syndromes and Immunosuppression. Lancet (2020) 395:1033–4. 10.1016/S0140-6736(20)30628-0 PMC7270045 32192578

[40] Lucas C Wong P Klein J Castro TBR Silva J Sundaram M. Longitudinal Analyses Reveal Immunological Misfiring in Severe COVID-19. Nature (2020) 584:463–9. 10.1038/s41586-020-2588-y PMC7477538 32717743

[41] Silvin A Chapuis N Dunsmore G Goubet AG Dubuisson A Derosa L. Elevated Calprotectin and Abnormal Myeloid Cell Subsets Discriminate Severe From Mild COVID-19. Cell (2020) 182:1401–18.e18. 10.1016/j.cell.2020.08.002 32810439 PMC7405878

[42] Schulte-Schrepping J Reusch N Paclik D Baßler K Schlickeiser S Zhang B. Severe COVID-19 Is Marked by a Dysregulated Myeloid Cell Compartment. Cell (2020) 182:1419–40.e23. 10.1016/j.cell.2020.08.001 32810438 PMC7405822

[43] Cao X. COVID-19: Immunopathology and its Implications for Therapy. Nat Rev Immunol (2020) 20:269–70. 10.1038/s41577-020-0308-3 PMC7143200 32273594

[44] Aschenbrenner AC Mouktaroudi M Krämer B Oestreich M Antonakos N Nuesch-Germano M. Disease Severity-Specific Neutrophil Signatures in Blood Transcriptomes Stratify COVID-19 Patients. Genome Med (2021) 13:7. 10.1186/s13073-020-00823-5 33441124 PMC7805430

[45] Liao M Liu Y Yuan J Wen Y Xu G Zhao J. Single-Cell Landscape of Bronchoalveolar Immune Cells in Patients With COVID-19. Nat Med (2020) 26:842–4. 10.1038/s41591-020-0901-9 32398875

[46] Siddiqi HK Mehra MR. COVID-19 Illness in Native and Immunosuppressed States: A Clinical–Therapeutic Staging Proposal. J Hear Lung Transplant (2020) 39:405–7. 10.1016/j.healun.2020.03.012 PMC7118652 32362390

[47] van Furth R Cohn ZA. The Origin and Kinetics of Mononuclear Phagocytes. J Exp Med (1968) 128:415–35. 10.1084/JEM.128.3.415 PMC2138527 5666958

[48] Bassler K Schulte-Schrepping J Warnat-Herresthal S Aschenbrenner AC Schultze JL. The Myeloid Cell Compartment-Cell by Cell. Annu Rev Immunol (2019) 37:269–93. 10.1146/annurev-immunol-042718-041728 30649988

[49] Kapellos TS Bonaguro L Gemünd I Reusch N Saglam A Hinkley ER. Human Monocyte Subsets and Phenotypes in Major Chronic Inflammatory Diseases. Front Immunol (2019) 10:2035. 10.3389/fimmu.2019.02035 31543877 PMC6728754

[50] Guilliams M Mildner A Yona S. Developmental and Functional Heterogeneity of Monocytes. Immunity (2018) 49:595–613. 10.1016/j.immuni.2018.10.005 30332628

[51] Prussin AJ Garcia EB Marr LC. Total Concentrations of Virus and Bacteria in Indoor and Outdoor Air. Environ Sci Technol Lett (2015) 2:84–8. 10.1021/acs.estlett.5b00050 PMC4515362 26225354

[52] Hussell T Bell TJ. Alveolar Macrophages: Plasticity in a Tissue-Specific Context. Nat Rev Immunol (2014) 14:81–93. 10.1038/nri3600 24445666

[53] Franke-Ullmann G Pförtner C Walter P Steinmüller C Lohmann-Matthes ML Kobzik L. Characterization of Murine Lung Interstitial Macrophages in Comparison With Alveolar Macrophages *In Vitro*. J Immunol (1996) 157:3097–104. 8816420

[54] Neupane AS Willson M Chojnacki AK Vargas E Silva Castanheira F Morehouse C Carestia A. Patrolling Alveolar Macrophages Conceal Bacteria From the Immune System to Maintain Homeostasis. Cell (2020) 183:110–25.e11. 10.1016/j.cell.2020.08.020 32888431

[55] Schulz C Perdiguero EG Chorro L Szabo-Rogers H Cagnard N Kierdorf K. A Lineage of Myeloid Cells Independent of Myb and Hematopoietic Stem Cells. Science (2012) 335:86–90. 10.1126/science.1219179 22442384

[56] Guilliams M De Kleer I Henri S Post S Vanhoutte L De Prijck S. Alveolar Macrophages Develop From Fetal Monocytes That Differentiate Into Long-Lived Cells in the First Week of Life. via GM-CSF. J Exp Med (2013) 210:1977–92. 10.1084/jem.20131199 PMC3782041 24043763

[57] Tarling JD Lin HS Hsu S. Self-Renewal of Pulmonary Alveolar Macrophages: Evidence From Radiation Chimera Studies. J Leukoc Biol (1987) 42:443–6. 10.1002/jlb.42.5.443 3316460

[58] Sawyer RT Strausbauch PH Volkman A. Resident Macrophage Proliferation in Mice Depleted of Blood Monocytes by Strontium-89. Lab Investig (1982) 46:165–70. 6174824

[59] Golde DW Byers LA Finley TN. Proliferative Capacity of Human Alveolar Macrophage. Nature (1974) 247:373–5. 10.1038/247373a0 4817856

[60] Eguíluz-Gracia I Schultz HHL Sikkeland LIB Danilova E Holm AM Pronk CJH. Long-Term Persistence of Human Donor Alveolar Macrophages in Lung Transplant Recipients. Thorax (2016) 71:1006–11. 10.1136/thoraxjnl-2016-208292 27329043

[61] Hashimoto D Chow A Noizat C Teo P Beasley MB Leboeuf M. Tissue-Resident Macrophages Self-Maintain Locally Throughout Adult Life With Minimal Contribution From Circulating Monocytes. Immunity (2013) 38:792–804. 10.1016/j.immuni.2013.04.004 23601688 PMC3853406

[62] Thomas ED Ramberg RE Sale GE Sparkes RS Golde DW. Direct Evidence for a Bone Marrow Origin of the Alveolar Macrophage in Man. Science (1976) 192:1016–8. 10.1126/science.775638 775638

[63] Guilliams M van de Laar L. A Hitchhiker’s Guide to Myeloid Cell Subsets: Practical Implementation of a Novel Mononuclear Phagocyte Classification System. Front Immunol (2015) 6:406. 10.3389/fimmu.2015.00406 26322042 PMC4531301

[64] Joshi N Walter JM Misharin AV. Alveolar Macrophages. Cell Immunol (2018) 330:86–90. 10.1016/j.cellimm.2018.01.005 29370889

[65] Watanabe S Alexander M Misharin AV Budinger GRS. The Role of Macrophages in the Resolution of Inflammation. J Clin Invest (2019) 129:2619–28. 10.1172/JCI124615 PMC6597225 31107246

[66] Coleman MM Ruane D Moran B Dunne PJ Keane J Mills KHG. Alveolar Macrophages Contribute to Respiratory Tolerance by Inducing FoxP3 Expression in Naive T Cells. Am J Respir Cell Mol Biol (2013) 48:773–80. 10.1165/rcmb.2012-0263OC 23492186

[67] Soroosh P Doherty TA Duan W Mehta AK Choi H Adams YF. Lung-Resident Tissue Macrophages Generate Foxp3+ Regulatory T Cells and Promote Airway Tolerance. J Exp Med (2013) 210:775–88. 10.1084/jem.20121849 PMC3620360 23547101

[68] Lipscomb MF Lyons CR Nunez G Ball EJ Stastny P Vial W. Human Alveolar Macrophages: HLA-DR-Positive Macrophages That are Poor Stimulators of a Primary Mixed Leukocyte Reaction. J Immunol (1986) 136(2):497–504. 2934472

[69] Snelgrove RJ Goulding J Didierlaurent AM Lyonga D Vekaria S Edwards L. A Critical Function for CD200 in Lung Immune Homeostasis and the Severity of Influenza Infection. Nat Immunol (2008) 9:1074–83. 10.1038/ni.1637 18660812

[70] Janssen WJ McPhillips KA Dickinson MG Linderman DJ Morimoto K Xiao YQ. Surfactant Proteins A and D Suppress Alveolar Macrophage Phagocytosis *via* Interaction With Sirpα. Am J Respir Crit Care Med (2008) 178:158–67. 10.1164/rccm.200711-1661OC PMC2453510 18420961

[71] Zhang J Tachado SD Patel N Zhu J Imrich A Manfruelli P. Negative Regulatory Role of Mannose Receptors on Human Alveolar Macrophage Proinflammatory Cytokine Release *In Vitro* . J Leukoc Biol (2005) 78:665–74. 10.1189/jlb.1204699 16000387

[72] Ghosh S Gregory D Smith A Kobzik L. MARCO Regulates Early Inflammatory Responses Against Influenza: A Useful Macrophage Function With Adverse Outcome. Am J Respir Cell Mol Biol (2011) 45:1036–44. 10.1165/rcmb.2010-0349OC PMC3262690 21562316

[73] Gao X Dong Y Liu Z Niu B. Silencing of Triggering Receptor Expressed on Myeloid Cells-2 Enhances the Inflammatory Responses of Alveolar Macrophages to Lipopolysaccharide. Mol Med Rep (2013) 7:921–6. 10.3892/mmr.2013.1268 23314916

[74] Fernandez S Jose P Avdiushko MG Kaplan AM Cohen DA. Inhibition of IL-10 Receptor Function in Alveolar Macrophages by Toll-Like Receptor Agonists. J Immunol (2004) 172:2613–20. 10.4049/jimmunol.172.4.2613 14764735

[75] Morris DG Huang X Kaminski N Wang Y Shapiro SD Dolganov G. Loss of Integrin αvβ6-Mediated TGF-β Activation Causes Mmp 12-Dependent Emphysema. Nature (2003) 422:169–73. 10.1038/nature01413 12634787

[76] Gautier EL Chow A Spanbroek R Marcelin G Greter M Jakubzick C. Systemic Analysis of Pparγ in Mouse Macrophage Populations Reveals Marked Diversity in Expression With Critical Roles in Resolution of Inflammation and Airway Immunity. J Immunol (2012) 189:2614–24. 10.4049/jimmunol.1200495 PMC3537497 22855714

[77] Lambrecht BN. Alveolar Macrophage in the Driver’s Seat. Immunity (2006) 24:366–8. 10.1016/j.immuni.2006.03.008 16618595

[78] Steinmüller C Franke-Ullmann G Lohmann-Matthes ML Emmendörffer A. Local Activation of Nonspecific Defense Against a Respiratory Model Infection by Application of Interferon-γ: Comparison Between Rat Alveolar and Interstitial Lung Macrophages. Am J Respir Cell Mol Biol (2000) 22:481–90. 10.1165/ajrcmb.22.4.3336 10745029

[79] Trapnell BC Whitsett JA. GM-CSF Regulates Pulmonary Surfactant Homeostasis and Alveolar Macrophage-Mediated Innate Host Defense. Annu Rev Physiol (2002) 64:775–802. 10.1146/annurev.physiol.64.090601.113847 11826288

[80] Baharom F Rankin G Blomberg A Smed-Sörensen A. Human Lung Mononuclear Phagocytes in Health and Disease. Front Immunol (2017) 8:499. 10.3389/fimmu.2017.00499 28507549 PMC5410584

[81] Laskin DL Sunil VR Gardner CR Laskin JD. Macrophages and Tissue Injury: Agents of Defense or Destruction? Annu Rev Pharmacol Toxicol (2011) 51:267–88. 10.1146/annurev.pharmtox.010909.105812 PMC3670679 20887196

[82] Ortega-Gómez A Perretti M Soehnlein O. Resolution of Inflammation: An Integrated View. EMBO Mol Med (2013) 5:661–74. 10.1002/emmm.201202382 PMC3662311 23592557

[83] Fadok VA Bratton DL Konowal A Freed PW Westcott JY Henson PM. Macrophages That Have Ingested Apoptotic Cells *In Vitro* Inhibit Proinflammatory Cytokine Production Through Autocrine/Paracrine Mechanisms Involving TGF-β, PGE2, and PAF. J Clin Invest (1998) 101:890–8. 10.1172/JCI1112 PMC508637 9466984

[84] Fitzpatrick AM Holguin F Teague WG Brown LAS. Alveolar Macrophage Phagocytosis is Impaired in Children With Poorly Controlled Asthma. J Allergy Clin Immunol (2008) 121(6):1372–8. 10.1016/j.jaci.2008.03.008 PMC2442216 18417198

[85] Hodge S Hodge G Scicchitano R Reynolds PN Holmes M. Alveolar Macrophages From Subjects With Chronic Obstructive Pulmonary Disease are Deficient in Their Ability to Phagocytose Apoptotic Airway Epithelial Cells. Immunol Cell Biol (2003) 81:289–96. 10.1046/j.1440-1711.2003.t01-1-01170.x 12848850

[86] Vandivier RW Richens TR Horstmann SA DeCathelineau AM Ghosh M Reynolds SD. Dysfunctional Cystic Fibrosis Transmembrane Conductance Regulator Inhibits Phagocytosis of Apoptotic Cells With Proinflammatory Consequences. Am J Physiol - Lung Cell Mol Physiol (2009) 297(4):L677–86. 10.1152/ajplung.00030.2009 PMC2770781 19633071

[87] Morimoto K Janssen WJ Terada M. Defective Efferocytosis by Alveolar Macrophages in IPF Patients. Respir Med (2012) 106:1800–3. 10.1016/j.rmed.2012.08.020 PMC4030720 22999220

[88] Aoshiba K Tamaoki J Nagai A. Acute Cigarette Smoke Exposure Induces Apoptosis of Alveolar Macrophages. Am J Physiol - Lung Cell Mol Physiol (2001) 281(6):L1392–401. 10.1152/ajplung.2001.281.6.l1392 11704535

[89] Gleeson LE O’Leary SM Ryan D McLaughlin AM Sheedy FJ Keane J. Cigarette Smoking Impairs the Bioenergetic Immune Response to Mycobacterium Tuberculosis Infection. Am J Respir Cell Mol Biol (2018) 59:572–9. 10.1165/rcmb.2018-0162OC 29944387

[90] Sussan TE Gajghate S Thimmulappa RK Ma J Kim JH Sudini K. Exposure to Electronic Cigarettes Impairs Pulmonary Anti-Bacterial and Anti-Viral Defenses in a Mouse Model. PloS One (2015) 10:e0116861. 10.1371/journal.pone.0116861 25651083 PMC4317176

[91] Wallace WAH Gillooly M Lamb D. Intra-Alveolar Macrophage Numbers in Current Smokers and non-Smokers: A Morphometric Study of Tissue Sections. Thorax (1992) 47:437–40. 10.1136/thx.47.6.437 PMC463808 1496503

[92] Schneider C Nobs SP Heer AK Kurrer M Klinke G van Rooijen N. Alveolar Macrophages Are Essential for Protection From Respiratory Failure and Associated Morbidity Following Influenza Virus Infection. PloS Pathog (2014) 10:e1004053. 10.1371/journal.ppat.1004053 24699679 PMC3974877

[93] Kim HM Lee Y-W Lee K-J Kim HS Cho SW van Rooijen N. Alveolar Macrophages Are Indispensable for Controlling Influenza Viruses in Lungs of Pigs. J Virol (2008) 82:4265–74. 10.1128/jvi.02602-07 PMC2293066 18287245

[94] Yilla M Harcourt BH Hickman CJ McGrew M Tamin A Goldsmith CS. SARS-Coronavirus Replication in Human Peripheral Monocytes/Macrophages. Virus Res (2005) 107:93–101. 10.1016/j.virusres.2004.09.004 15567038 PMC7114182

[95] Smith MS Bentz GL Alexander JS Yurochko AD. Human Cytomegalovirus Induces Monocyte Differentiation and Migration as a Strategy for Dissemination and Persistence. J Virol (2004) 78:4444–53. 10.1128/jvi.78.9.4444-4453.2004 PMC387677 15078925

[96] Nottet HS Persidsky Y Sasseville VG Nukuna AN Bock P Zhai QH. Mechanisms for the Transendothelial Migration of HIV-1-Infected Monocytes Into Brain. J Immunol (1996) 156:1284–95. 8558009

[97] Desforges M Miletti TC Gagnon M Talbot PJ. Activation of Human Monocytes After Infection by Human Coronavirus 229E. Virus Res (2007) 130:228–40. 10.1016/j.virusres.2007.06.016 PMC7114174 17669539

[98] Al-Qahtani AA Lyroni K Aznaourova M Tseliou M Al-Anazi MR Al-Ahdal MN. Middle East Respiratory Syndrome Corona Virus Spike Glycoprotein Suppresses Macrophage Responses *via* DPP4-Mediated Induction of IRAK-M and Pparγ. Oncotarget (2017) 8:9053–66. 10.18632/oncotarget.14754 PMC5354714 28118607

[99] Nikitina E Larionova I Choinzonov E Kzhyshkowska J. Monocytes and Macrophages as Viral Targets and Reservoirs. Int J Mol Sci (2018) 19:2821. 10.3390/ijms19092821 PMC6163364 30231586

[100] Cheung CY Poon LLM Ng IHY Luk W Sia S-F Wu MHS. Cytokine Responses in Severe Acute Respiratory Syndrome Coronavirus-Infected Macrophages In Vitro: Possible Relevance to Pathogenesis. J Virol (2005) 79:7819–26. 10.1128/jvi.79.12.7819-7826.2005 PMC1143636 15919935

[101] Wang J Nikrad MP Travanty EA Zhou B Phang T Gao B. Innate Immune Response of Human Alveolar Macrophages During Influenza a Infection. PloS One (2012) 7:e29879. 10.1371/journal.pone.0029879 22396727 PMC3292548

[102] Kumagai Y Takeuchi O Kato H Kumar H Matsui K Morii E. Alveolar Macrophages Are the Primary Interferon-α Producer in Pulmonary Infection With RNA Viruses. Immunity (2007) 27:240–52. 10.1016/j.immuni.2007.07.013 17723216

[103] Wang J Nikrad MP Phang T Gao B Alford T Ito Y. Innate Immune Response to Influenza A Virus in Differentiated Human Alveolar Type II Cells. Am J Respir Cell Mol Biol (2011) 45:582–91. 10.1165/rcmb.2010-0108OC PMC3175576 21239608

[104] Wong MT Chen SSL. Emerging Roles of Interferon-Stimulated Genes in the Innate Immune Response to Hepatitis C Virus Infection. Cell Mol Immunol (2016) 13:11–35. 10.1038/cmi.2014.127 25544499 PMC4712384

[105] Hambleton S Goodbourn S Young DF Dickinson P Mohamad SMB Valappil M. STAT2 Deficiency and Susceptibility to Viral Illness in Humans. Proc Natl Acad Sci USA (2013) 110:3053–8. 10.1073/pnas.1220098110 PMC3581986 23391734

[106] Joel Funk C Wang J Ito Y Travanty EA Voelker DR Holmes KV. Infection of Human Alveolar Macrophages by Human Coronavirus Strain 229E. J Gen Virol (2012) 93:494–503. 10.1099/vir.0.038414-0 22090214 PMC3352353

[107] Califano D Furuya Y Metzger DW. Effects of Influenza on Alveolar Macrophage Viability Are Dependent on Mouse Genetic Strain. J Immunol (2018) 201:134–44. 10.4049/jimmunol.1701406 PMC6008236 29760191

[108] Arunachalam PS Wimmers F Mok CKP Perera RAPM Scott M Hagan T. Systems Biological Assessment of Immunity to Mild Versus Severe COVID-19 Infection in Humans. Science (2020) 369:1210–20. 10.1126/SCIENCE.ABC6261 PMC7665312 32788292

[109] Hadjadj J Yatim N Barnabei L Corneau A Boussier J Smith N. Impaired Type I Interferon Activity and Inflammatory Responses in Severe COVID-19 Patients. Science (2020) 369:718–24. 10.1126/science.abc6027 PMC7402632 32661059

[110] Zhou Z Ren L Zhang L Zhong J Xiao Y Jia Z. Heightened Innate Immune Responses in the Respiratory Tract of COVID-19 Patients. Cell Host Microbe (2020) 27:883–90.e2. 10.1016/j.chom.2020.04.017 32407669 PMC7196896

[111] Fajgenbaum DC June CH. Cytokine Storm. N Engl J Med (2020) 383:2255–73. 10.1056/nejmra2026131 PMC7727315 33264547

[112] Kox M Waalders NJB Kooistra EJ Gerretsen J Pickkers P. Cytokine Levels in Critically Ill Patients With COVID-19 and Other Conditions. JAMA - J Am Med Assoc (2020) 324:1565–7. 10.1001/jama.2020.17052 PMC7489366 32880615

[113] Monneret G Benlyamani I Gossez M Bermejo-Martin JF Martín-Fernandez M Sesques P. COVID-19: What Type of Cytokine Storm Are We Dealing With? J Med Virol (2021) 93:197–8. 10.1002/jmv.26317 PMC7405026 32681651

[114] Sinha P Matthay MA Calfee CS. Is a “Cytokine Storm” Relevant to COVID-19? JAMA Intern Med (2020) 180:1152–4. 10.1001/jamainternmed.2020.3313 32602883

[115] Leisman DE Ronner L Pinotti R Taylor MD Sinha P Calfee CS. Cytokine Elevation in Severe and Critical COVID-19: A Rapid Systematic Review, Meta-Analysis, and Comparison With Other Inflammatory Syndromes. Lancet Respir Med (2020) 8:1233–44. 10.1016/S2213-2600(20)30404-5 PMC7567529 33075298

[116] Khanmohammadi S Rezaei N. Role of Toll-Like Receptors in the Pathogenesis of COVID-19. J Med Virol (2021) 93:2735–9. 10.1002/jmv.26826 PMC8014260 33506952

[117] Yang D Geng T Harrison AG Wang P. Differential Roles of RIG-I-Like Receptors in SARS-CoV-2 Infection. bioRxiv Prepr Serv Biol (2021). 10.1101/2021.02.10.430677 PMC8421188 34488908

[118] Zhao Y Kuang M Li J Zhu L Jia Z Guo X. SARS-CoV-2 Spike Protein Interacts With and Activates TLR4. Cell Res (2021) 31:818–20. 10.1038/s41422-021-00495-9 PMC7975240 33742149

[119] Zheng M Karki R Williams EP Yang D Fitzpatrick E Vogel P. TLR2 Senses the SARS-CoV-2 Envelope Protein to Produce Inflammatory Cytokines. Nat Immunol (2021) 22:829–38. 10.1038/s41590-021-00937-x PMC8882317 33963333

[120] Thépaut M Luczkowiak J Vivès C Labiod N Bally I Lasala F. Dc/L-SIGN Recognition of Spike Glycoprotein Promotes SARS-CoV-2 Trans-Infection and can be Inhibited by a Glycomimetic Antagonist. PloS Pathog (2021) 17:e1009576. 10.1371/journal.ppat.1009576 34015061 PMC8136665

[121] Lu Q Liu J Zhao S Gomez Castro MF Laurent-Rolle M Dong J. SARS-CoV-2 Exacerbates Proinflammatory Responses in Myeloid Cells Through C-Type Lectin Receptors and Tweety Family Member 2. Immunity (2021) 54:1304–1319.e9. 10.1016/j.immuni.2021.05.006 34048708 PMC8106883

[122] Yang D Chu H Hou Y Chai Y Shuai H Lee ACY. Attenuated Interferon and Proinflammatory Response in SARS-CoV-2-Infected Human Dendritic Cells is Associated With Viral Antagonism of STAT1 Phosphorylation. J Infect Dis (2020) 222:734–45. 10.1093/infdis/jiaa356 PMC7337793 32563187

[123] Zheng J Wang Y Li K Meyerholz DK Allamargot C Perlman S. Severe Acute Respiratory Syndrome Coronavirus 2-Induced Immune Activation and Death of Monocyte-Derived Human Macrophages and Dendritic Cells. J Infect Dis (2021) 223:785–95. 10.1093/infdis/jiaa753 PMC7799009 33277988

[124] Chu H Chan JFW Wang Y Yuen TTT Chai Y Hou Y. Comparative Replication and Immune Activation Profiles of SARS-CoV-2 and SARS-CoV in Human Lungs: An Ex Vivo Study With Implications for the Pathogenesis of COVID-19. Clin Infect Dis (2020) 71:1400–9. 10.1093/cid/ciaa410 PMC7184390 32270184

[125] Martines RB Ritter JM Matkovic E Gary J Bollweg BC Bullock H. Pathology and Pathogenesis of SARS-CoV-2 Associated With Fatal Coronavirus Disease, United States. Emerg Infect Dis (2020) 26:2005–15. 10.3201/eid2609.202095 PMC7454055 32437316

[126] Lv J Wang Z Qu Y Zhu H Zhu Q Tong W. Distinct Uptake, Amplification, and Release of SARS-CoV-2 by M1 and M2 Alveolar Macrophages. Cell Discovery (2021) 7:24. 10.1038/s41421-021-00258-1 33850112 PMC8043100

[127] Pantazi I Al-Qahtani AA Alhamlan FS Alothaid H Matou-Nasri S Sourvinos G. SARS-CoV-2/ACE2 Interaction Suppresses IRAK-M Expression and Promotes Pro-Inflammatory Cytokine Production in Macrophages. Front Immunol (2021) 12:683800. 10.3389/FIMMU.2021.683800 34248968 PMC8261299

[128] Liu L Wei Q Lin Q Fang J Wang H Kwok H. Anti-Spike IgG Causes Severe Acute Lung Injury by Skewing Macrophage Responses During Acute SARS-CoV Infection. JCI Insight (2019) 4:e123158. 10.1172/jci.insight.123158 PMC6478436 30830861

[129] Jaume M Yip MS Cheung CY Leung HL Li PH Kien F. Anti-Severe Acute Respiratory Syndrome Coronavirus Spike Antibodies Trigger Infection of Human Immune Cells *via* a pH- and Cysteine Protease-Independent Fc R Pathway. J Virol (2011) 85:10582–97. 10.1128/jvi.00671-11 PMC3187504 21775467

[130] Codo AC Davanzo GG Monteiro L de B de Souza GF Muraro SP Virgilio-da-Silva JV. Elevated Glucose Levels Favor SARS-CoV-2 Infection and Monocyte Response Through a HIF-1α/Glycolysis-Dependent Axis. Cell Metab (2020) 32:437–46.e5. 10.1016/j.cmet.2020.07.007 32697943 PMC7367032

[131] da Silva Gomes Dias S Soares VC Ferreira AC Sacramento CQ Fintelman-Rodrigues N Temerozo JR. Lipid Droplets Fuel SARS-CoV-2 Replication and Production of Inflammatory Mediators. PloS Pathog (2020) 16:e1009127. 10.1371/journal.ppat.1009127 33326472 PMC7773323

[132] Zhou Y Fu B Zheng X Wang D Zhao C Qi Y. Pathogenic T-Cells and Inflammatory Monocytes Incite Inflammatory Storms in Severe COVID-19 Patients. Natl Sci Rev (2020) 7:998–1002. 10.1093/nsr/nwaa041 PMC7108005 34676125

[133] Laing AG Lorenc A del Molino del Barrio I Das A Fish M Monin L. A Dynamic COVID-19 Immune Signature Includes Associations With Poor Prognosis. Nat Med (2020) 26:1623–35. 10.1038/s41591-020-1038-6 32807934

[134] Payen D Cravat M Maadadi H Didelot C Prosic L Dupuis C. A Longitudinal Study of Immune Cells in Severe COVID-19 Patients. Front Immunol (2020) 11:580250. 10.3389/fimmu.2020.580250 33178207 PMC7597438

[135] Gatti A Radrizzani D Viganò P Mazzone A Brando B. Decrease of Non-Classical and Intermediate Monocyte Subsets in Severe Acute SARS-CoV-2 Infection. Cytom Part A (2020) 97:887–90. 10.1002/cyto.a.24188 PMC7404377 32654350

[136] Ferreira AC Soares VC de Azevedo-Quintanilha IG Dias S da SG Fintelman-Rodrigues N Sacramento CQ. SARS-CoV-2 Engages Inflammasome and Pyroptosis in Human Primary Monocytes. Cell Death Discov (2021) 7:43. 10.1038/s41420-021-00428-w 33649297 PMC7919254

[137] Schultze JL Mass E Schlitzer A. Emerging Principles in Myelopoiesis at Homeostasis and During Infection and Inflammation. Immunity (2019) 50:288–301. 10.1016/j.immuni.2019.01.019 30784577

[138] Mann ER Menon M Knight SB Konkel JE Jagger C Shaw TN. Longitudinal Immune Profiling Reveals Key Myeloid Signatures Associated With COVID-19. Sci Immunol (2020) 5:eabd6197. 10.1126/SCIIMMUNOL.ABD6197 32943497 PMC7857390

[139] Giamarellos-Bourboulis EJ Netea MG Rovina N Akinosoglou K Antoniadou A Antonakos N. Complex Immune Dysregulation in COVID-19 Patients With Severe Respiratory Failure. Cell Host Microbe (2020) 27:992–1000.e3. 10.1016/j.chom.2020.04.009 32320677 PMC7172841

[140] Spinetti T Hirzel C Fux M Walti LN Schober P Stueber F. Reduced Monocytic Human Leukocyte Antigen-DR Expression Indicates Immunosuppression in Critically Ill COVID-19 Patients. Anesth Analg (2020) 131:993–9. 10.1213/ANE.0000000000005044 PMC7288784 32925314

[141] Wang F Hou H Yao Y Wu S Huang M Ran X. Systemically Comparing Host Immunity Between Survived and Deceased COVID-19 Patients. Cell Mol Immunol (2020) 17:875–7. 10.1038/s41423-020-0483-y PMC7295144 32541836

[142] Kvedaraite E Hertwig L Sinha I Ponzetta A Myrberg IH Lourda M. Major Alterations in the Mononuclear Phagocyte Landscape Associated With COVID-19 Severity. Proc Natl Acad Sci USA (2021) 118(6):e2018587118. 10.1073/pnas.2018587118 33479167 PMC8017719

[143] Carter MJ Fish M Jennings A Doores KJ Wellman P Seow J. Peripheral Immunophenotypes in Children With Multisystem Inflammatory Syndrome Associated With SARS-CoV-2 Infection. Nat Med (2020) 26:1701–7. 10.1038/s41591-020-1054-6 32812012

[144] Hegde S Leader AM Merad M. MDSC: Markers, Development, States, and Unaddressed Complexity. Immunity (2021) 54:875–84. 10.1016/j.immuni.2021.04.004 PMC8709560 33979585

[145] Falck-Jones S Vangeti S Yu M Falck-Jones R Cagigi A Badolati I. Functional Monocytic Myeloid-Derived Suppressor Cells Increase in Blood But Not Airways and Predict COVID-19 Severity. J Clin Invest (2021) 131(6):e144734. 10.1172/JCI144734 PMC7954608 33492309

[146] Thompson EA Cascino K Ordonez AA Zhou W Vaghasia A Hamacher-Brady A. Metabolic Programs Define Dysfunctional Immune Responses in Severe COVID-19 Patients. Cell Rep (2021) 34:108863. 10.1016/j.celrep.2021.108863 33691089 PMC7908880

[147] Wen W Su W Tang H Le W Zhang X Zheng Y. Immune Cell Profiling of COVID-19 Patients in the Recovery Stage by Single-Cell Sequencing. Cell Discov (2020) 6:6. 10.1038/s41421-020-0168-9 32377375 PMC7197635

[148] Lee JS Park S Jeong HW Ahn JY Choi SJ Lee H. Immunophenotyping of Covid-19 and Influenza Highlights the Role of Type I Interferons in Development of Severe Covid-19. Sci Immunol (2020) 5:1554. 10.1126/sciimmunol.abd1554 PMC7402635 32651212

[149] Szabo PA Dogra P Gray JI Wells SB Connors TJ Weisberg SP. Longitudinal Profiling of Respiratory and Systemic Immune Responses Reveals Myeloid Cell-Driven Lung Inflammation in Severe COVID-19. Immunity (2021) 54:797–814.e6. 10.1016/j.immuni.2021.03.005 33765436 PMC7951561

[150] Stephenson E Reynolds G Botting RA Calero-Nieto FJ Morgan MD Tuong ZK. Single-Cell Multi-Omics Analysis of the Immune Response in COVID-19. Nat Med (2021) 27:904–16. 10.1038/s41591-021-01329-2 PMC8121667 33879890

[151] Su Y Chen D Yuan D Lausted C Choi J Dai CL. Multi-Omics Resolves a Sharp Disease-State Shift Between Mild and Moderate COVID-19. Cell (2020) 183:1479–95.e20. 10.1016/j.cell.2020.10.037 33171100 PMC7598382

[152] Cao Y Su B Guo X Sun W Deng Y Bao L. Potent Neutralizing Antibodies Against SARS-CoV-2 Identified by High-Throughput Single-Cell Sequencing of Convalescent Patients’ B Cells. Cell (2020) 182:73–84.e16. 10.1016/j.cell.2020.05.025 32425270 PMC7231725

[153] Melms JC Biermann J Huang H Wang Y Nair A Tagore S. A Molecular Single-Cell Lung Atlas of Lethal COVID-19. Nature (2021) 33:15. 10.1038/s41586-021-03569-1 PMC8814825 33915568

[154] Wauters E Van Mol P Garg AD Jansen S Van Herck Y Vanderbeke L. Discriminating Mild From Critical COVID-19 by Innate and Adaptive Immune Single-Cell Profiling of Bronchoalveolar Lavages. Cell Res (2021) 31:272–90. 10.1038/s41422-020-00455-9 PMC8027624 33473155

[155] Wilk AJ Rustagi A Zhao NQ Roque J Martínez-Colón GJ McKechnie JL. A Single-Cell Atlas of the Peripheral Immune Response in Patients With Severe COVID-19. Nat Med (2020) 26:1070–6. 10.1038/s41591-020-0944-y PMC7382903 32514174

[156] Bernardes JP Mishra N Tran F Rosenstiel P. Longitudinal Multi-Omics Analyses Identify Responses of Megakaryocytes, Erythroid Cells, and Plasmablasts as Hallmarks of Severe COVID-19. Immunity (2020) 53:1296–314.e9. 10.1016/j.immuni.2020.11.017 33296687 PMC7689306

[157] Zhang JY Wang XM Xing X Xu Z Zhang C Song JW. Single-Cell Landscape of Immunological Responses in Patients With COVID-19. Nat Immunol (2020) 21:1107–18. 10.1038/s41590-020-0762-x 32788748

[158] Grant RA Morales-Nebreda L Markov NS Swaminathan S Querrey M Guzman ER. Circuits Between Infected Macrophages and T Cells in SARS-CoV-2 Pneumonia. Nature (2021) 590:635–41. 10.1038/s41586-020-03148-w PMC7987233 33429418

[159] Combes AJ Courau T Kuhn NF Hu KH Ray A Chen WS. Global Absence and Targeting of Protective Immune States in Severe COVID-19. Nature (2021) 591:124–30. 10.1038/s41586-021-03234-7 PMC8567458 33494096

[160] Nouailles G Wyler E Pennitz P Postmus D Kazmierski J Pott F. Longitudinal Omics in Syrian Hamsters Integrated With Human Data Unravel Complexity 1 of Moderate Immune Responses to SARS-CoV-2 2 3. bioRxiv (2020) 2020:12. 10.1101/2020.12.18.423524

[161] Sánchez-Cerrillo I Landete P Aldave B Sánchez-Alonso S Sánchez-Azofra A Marcos-Jiménez A. COVID-19 Severity Associates With Pulmonary Redistribution of CD1c+ DCs and Inflammatory Transitional and Nonclassical Monocytes. J Clin Invest (2020) 130:6290–300. 10.1172/JCI140335 PMC7685723 32784290

[162] Bost P Giladi A Liu Y Bendjelal Y Xu G David E. Host-Viral Infection Maps Reveal Signatures of Severe COVID-19 Patients. Cell (2020) 181:1475–1488.e12. 10.1016/j.cell.2020.05.006 32479746 PMC7205692

